# THE VALUE OF COMMUNITY–UNIVERSITY PARTNERSHIPS IN GHANA: IMPLEMENTING CULTURALLY RESPONSIVE DEMENTIA EDUCATION

**DOI:** 10.1093/geroni/igae098.1875

**Published:** 2024-12-31

**Authors:** Janelle Gore, Fayron Epps

**Affiliations:** University of Alabama, Alabama, United States; The University of Texas Health San Antonio, San Antonio, Texas, United States

## Abstract

Alzheimer’s disease and related dementias have not been given much attention in sub-Saharan Africa. Although the prevalence of dementia in Ghana is not yet established, those who present cognitive impairment are often stigmatized, discriminated against, or physically harmed by community members. The mistreatment may be due to a lack of dementia knowledge and awareness in Ghana. Recognizing the critical need to increase dementia knowledge and awareness in Ghana, Alzheimer’s Ghana (Ghana), AlterDementia (US), and researchers from the University of Georgia and Emory University (US) partnered to develop and implement a culturally adapted and relevant dementia education intervention. Through a series of collaborative sessions, both organizations and researchers co-created educational materials and intervention strategies addressing dementia awareness and knowledge. Education sessions consisted of a dementia overview, brain health overview, community testimonies, local resources, question and answer session, and closing remarks. Community-based participatory research principles were used to engage public officials, faith-based organizations, television personalities, healthcare professionals, secondary school educators, and researchers in Ghana to facilitate nine dementia education sessions, spanning 15-90 minutes, to over 800 participants across four regions of Ghana. Surveys and field notes were used to collect demographic and dementia knowledge and awareness data. Preliminary findings suggest promising avenues for improving dementia knowledge and reducing stigma. This work underscores the value of community-university partnerships to culturally adapt evidence-based interventions to advance ongoing community initiatives addressing dementia.

